# A method for predicting student psychological health based on behavioral time series analysis

**DOI:** 10.3389/fpsyt.2026.1773886

**Published:** 2026-04-28

**Authors:** Miao Hao

**Affiliations:** School of Literature and Journalism, Xihua University, Chengdu, Sichuan, China

**Keywords:** Apriori algorithm, attention mechanism, behavioral time series analysis, Jenks natural breaks algorithm, psychological health prediction

## Abstract

**Background:**

Early identification of student psychological issues is essential for providing timely support and preventing safety incidents. However, distinguishing between normal behavioral variability and critical indicators of distress within complex, high-dimensional campus data remains a significant challenge. This study proposes a sensitive temporal modeling approach designed to detect abnormal behavioral patterns and predict psychological health states.

**Methods:**

We developed a two-phase methodology to enhance feature quality and temporal sensitivity. First, the Jenks natural breaks algorithm was employed for optimal feature discretization to manage data heterogeneity. Subsequently, the Apriori algorithm was utilized to perform association rule mining, filtering for behavioral features with the strongest correlations to specific psychological states. Finally, we implemented a gated module enhanced by attention mechanisms to model historical behavioral time series. This module was specifically designed to integrate long-term habits with short-term fluctuations, dynamically assigning higher weights to irregular behavioral changes.

**Results:**

The model was evaluated using the public StudentLife dataset. Comparative experiments demonstrate that the proposed method significantly outperforms several baseline models across four key evaluation metrics. The results indicate that the attention-enhanced gated module effectively captures the temporal evolution of mental health states by prioritizing discriminative behavioral anomalies.

**Conclusions:**

Our findings validate the efficacy of combining association rule mining with attention-based temporal modeling for psychological health prediction. This approach offers a practical, precise tool for campus administrators to identify students at risk, enabling proactive intervention through the analysis of complex behavioral data.

## 1 Introduction

The psychological well-being of students is a cornerstone of their academic success and overall life trajectory. Poor psychological health not only destabilizes an individual’s emotional and behavioral patterns but can also precipitate severe safety incidents. Consequently, the early identification of psychological distress and the timely provision of intervention are paramount. Such proactive measures are essential for preventing potential crises before they escalate Furukawa (1) Hayakawa (2) Hayakawa (2) Iwata et al. (3). Currently, educational institutions predominantly rely on periodic self-report surveys to assess students’ mental states. However, this approach suffers from inherent temporal limitations. Surveys are typically administered at sparse intervals—such as the start of an academic year—and often target students who already exhibit visible signs of distress. This reactive mechanism fails to provide continuous monitoring and lacks the capacity to issue early warnings for students in the nascent stages of psychological risk Cohen et al. (4) Yan (5).

From a psychological perspective, latent mental states are often externalized through observable behavioral patterns. This intrinsic link suggests that analyzing behavioral trajectories offers a viable pathway for real-time psychological health monitoring. While diverse data sources exist for capturing student behavior—including campus administrative systems, mobile sensors, and institutional logs—each presents distinct tradeoffs between ecological validity, data granularity, and privacy protection. While the proliferation of mobile sensors has enabled researchers to track personal activities, such as physical movements and social interactions Muro et al. (6) Im Jin and Kim (7) Trpcevska (8), such approaches offer high temporal resolution and naturalistic capture of behavior *in situ*. However, implementation of behavioral monitoring systems necessitates careful consideration of privacy-preserving mechanisms, informed consent protocols, and ethical deployment frameworks to safeguard student autonomy Huang et al. (9).

In recent years, the application of deep learning to student behavior modeling has garnered widespread attention, significantly enhancing the representation of complex behavioral sequences. For instance, researchers have introduced hypergraphs and cascade attention transformer (CAT) modules to capture high-order relationships and weight different behaviors Li et al. (10) Winata et al. (11). To address temporal dynamics, context-aware Long Short-Term Memory (LSTM) networks have been employed to fuse personal and social information for multitask learning Winata et al. (11) Chen and Liu (12). Furthermore, Spatio-Temporal Graph Convolutional Networks (ST-GCN) have been utilized to model the spatial semantics of campus activities Zhou et al. (13), while local-global heterogeneous graph models have been devised to aggregate short-term behavioral fluctuations Vihavainen et al. (14) Breiman (15) Burges (16). More recently, deep-trained tree-based gated neural networks have been proposed to overcome gradient vanishing issues in long-sequence modeling LeCun et al. (17).

Despite these methodological innovations, a critical gap remains in the literature: most existing studies treat student behaviors as homogeneous sequences, often overlooking the specific correlation between abnormal behavioral patterns and psychological crises Sakai and Noguchi (18) Cuartero and Tur (19) McEown et al. (20). Standard models tend to focus on general behavioral trends, potentially smoothing out sudden, irregular anomalies that are actually the most significant indicators of mental distress. Additionally, while attention-based temporal models have been widely applied in sequential tasks, their specific adaptation to behavioral anomaly detection in mental health contexts warrants further investigation.

To bridge this gap, this paper proposes a sensitive method for modeling student behavior time series, specifically tailored to detect abnormal behaviors and predict psychological health. Our approach is designed to capture the subtle deviations in student activities that signal underlying distress. First, to address the noise and heterogeneity in raw behavioral data, we employ a rigorous feature engineering strategy. We utilize the Jenks natural breaks algorithm do Carvalhal Monteiro et al. (21) to optimally discretize continuous feature data, followed by the Apriori algorithm Yan (5) to mine association rules. This process filters out irrelevant noise and identifies specific behavioral features that exhibit strong correlations with psychological health states. Second, we introduce a temporal modeling framework (rather than a fundamentally new architecture) that integrates attention mechanisms with a gated module. Unlike traditional recurrent networks, our model is designed to be highly sensitive to historical anomalies. By dynamically assigning greater weight to psychologically sensitive behavioral changes and fusing long-term habits with short-term fluctuations, our method learns a more discriminative representation of student behavior. This synergistic approach effectively minimizes the omission of critical risk indicators, thereby enhancing the precision and reliability of psychological health predictions.

## 2 Multidimensional student behavioral feature extraction and feature association analysis

The conceptual feature family used in this section (regularity, morning activation, and social proximity) originates from prior campus behavior analytics that quantified orderliness and diligence in institutional data Cao et al. (22). In the present study, we retain this conceptual structure but operationalize each feature directly from StudentLife mobile sensing signals (accelerometer, microphone, and Wi-Fi).

**Figure 1:**
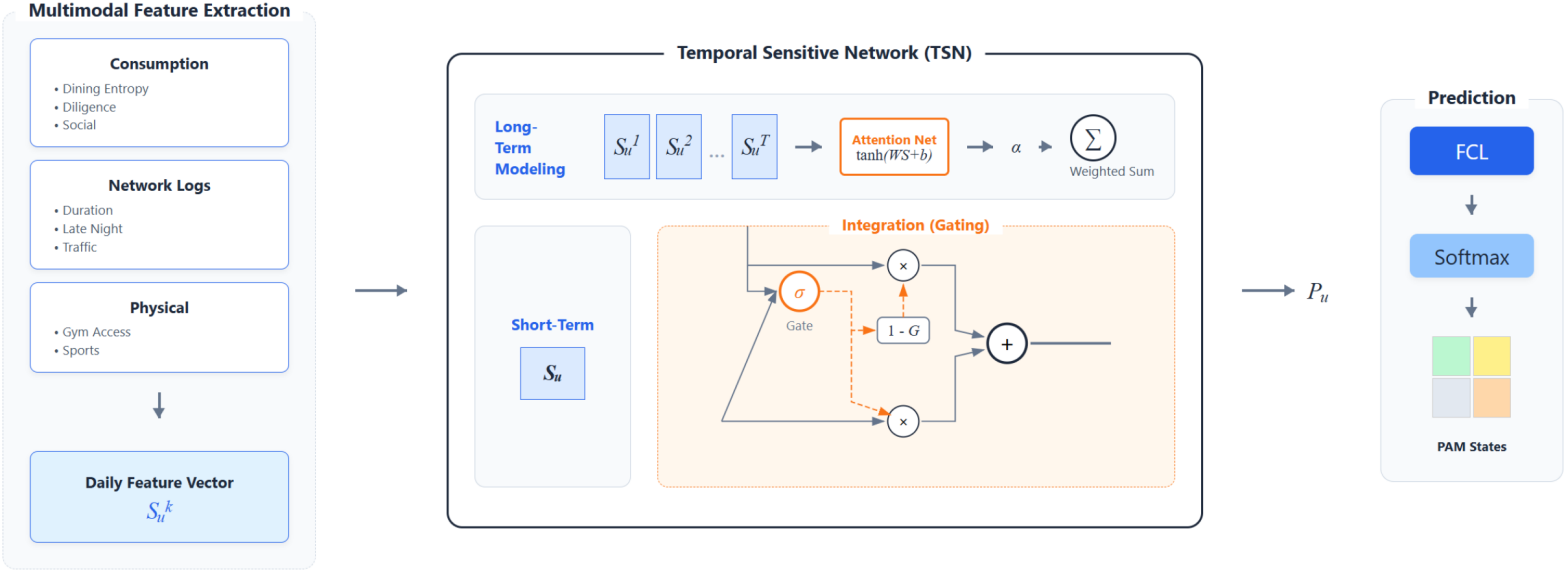
The architecture of the temporal sensitive network (TSN). (Left) Multimodal features are extracted from daily records. (Center) The model fuses long-term habits and short-term behaviors via a Gating Mechanism. (Right) The fused representation predicts psychological states.

This study leverages a comprehensive, high-granularity dataset to conduct a deep analysis of daily behavioral patterns, as shown in **Figure 1**. Accordingly, all feature definitions below are instantiated with mobile sensing observations: accelerometer states for physical routine regularity, Wi-Fi traces for mobility structure and co-location, and microphone-derived acoustic context for social activity intensity. This signal-level mapping preserves methodological continuity with the original framework while ensuring direct consistency with the data used in experiments.

### 2.1 Extraction of regularity, diligence, and social interaction features

Given the established psychometric evidence linking lifestyle irregularity to mental health challenges such as anxiety and depression Sano et al. (23, 24); Tate et al. (25), we extract three behavior constructs from StudentLife sensors: temporal regularity, morning activation (diligence proxy), and social interaction intensity.

#### 2.1.1 Quantification of daily regularity via sensor-state entropy

Irregular daily routines are early indicators of psychological distress. To quantify the stability of each student’s daily rhythm, we compute entropy over sensor-derived activity events. The daily timeline (06:00 to 22:00) is discretized into *n* = 48 intervals (30 minutes each), and each interval is assigned event counts from non-stationary accelerometer states (walking/running) and Wi-Fi location transitions. Students with stable routines exhibit concentrated temporal distributions (low entropy), whereas highly irregular routines exhibit dispersed distributions (high entropy). The regularity score, *R_f_*, is defined as:

(1) $$R_{f}=\frac{T_{d}}{D}(-\sum_{i=1}^{n}p_{i}\log\;p_{i})$$

where *D* denotes the number of valid sensing days (at least 6 hours of combined accelerometer and Wi-Fi coverage), and *T_d_*is the total observation span in days. The factor $\frac{T_{d}}{D}$ penalizes sparse participation. *p_i_*represents the probability of a sensor-derived behavioral event in interval *i*, computed as the event count in interval *i* divided by the total event count across all intervals.

#### 2.1.2 Diligence assessment via morning activity onset

The timing of first sustained daily activity is strongly associated with morningness and sleep hygiene. In StudentLife, we define daily onset using sensor evidence rather than transaction records: the earliest timestamp at which either (i) accelerometer states indicate continuous non-stationary activity for at least 5 minutes, or (ii) Wi-Fi traces indicate the first transition away from the nighttime residence cluster. The diligence metric, $R_{wh}$, is calculated as the average onset time, as shown in Equation 2:

(2) $$R_{wh}=\frac{1}{T}\sum_{j=1}^{T}t_{j}$$

where *T* is the number of days with valid morning sensing records, and *t_j_*is the normalized Unix timestamp of the first sustained activity event on day *j*. A lower $R_{wh}$indicates an earlier start to the day, corresponding to higher behavioral diligence.

#### 2.1.3 Social interaction via Wi-Fi co-location and acoustic context

Social isolation is a major risk factor for psychological deterioration. We therefore infer social interaction episodes from multimodal co-evidence: (a) interpersonal proximity from overlapping Wi-Fi location segments, and (b) conversational context from microphone state labels (*Speaking*/*Noisy*). A social episode is counted when two participants share a location overlap within a short temporal window and at least one participant exhibits conversational acoustic states during that overlap.

To ensure robustness, we apply strict filtering. 1. Exclusion of low-fidelity intervals: windows dominated by *Unknown* microphone labels or fewer than three Wi-Fi scans are removed. 2. Coverage constraint: student-days with less than 6 hours of valid sensing coverage are excluded to reduce sparsity bias.

The procedure for quantifying social interactions is formally described in **Algorithm 1**.

Algorithm 1

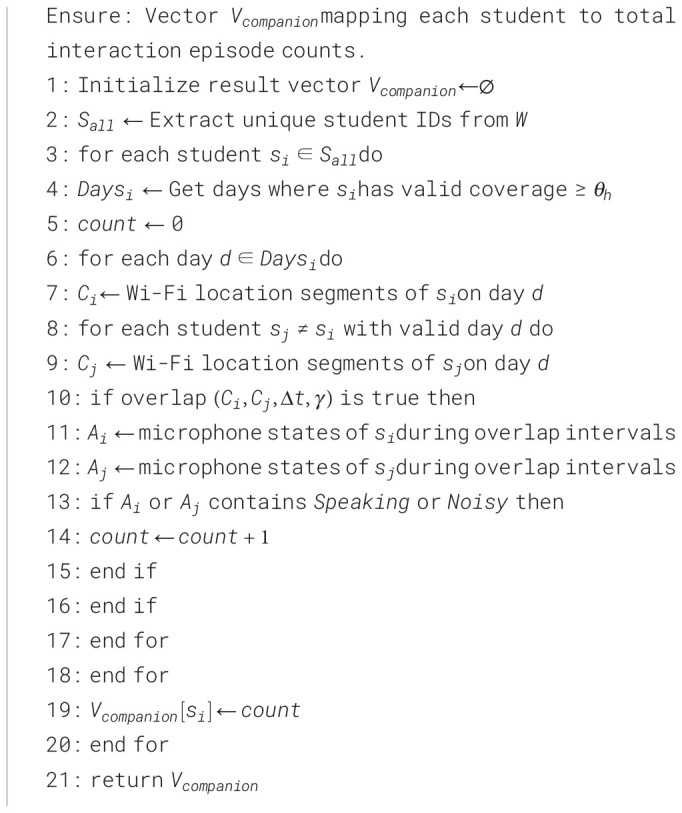

### 2.2 Extraction of student internet usage features

Digital behavior patterns, particularly excessive or irregular internet usage, are frequently associated with sleep displacement, academic procrastination, and social withdrawal. In mobile sensing contexts, the following metrics have direct analogs: usage duration (*R_dt_*) corresponds to screen-on time and session length from phone logs; late-night activity (*R_nt_*) to last device unlock or app usage timestamp; and data consumption (*R_fr_*) to cellular or Wi-Fi data traffic. We extract three key metrics to capture these digital habits, distinguishing between weekdays (academic focus) and weekends (leisure focus).

1. Average Usage Duration (*R_dt_*): This metric quantifies the time a student spends online. We calculate the mean duration of internet sessions separately for weekdays and weekends to differentiate between study-related research and potential leisure addiction.

(3) $$R_{dt}=\frac{1}{T_{d}}\sum_{i=1}^{K}\text{Δ}t_{i}$$

As shown in Equation 3, where *K* is the total number of internet sessions, $\text{Δ}t_{i}$ is the duration of the *i*-th session, and *T_d_*is the number of observed days.

2. Late-Night Activity (*R_nt_*): The timestamp of the last logout is a critical indicator of sleep onset. To accurately capture “night owl” behavior, we employ a logical day shift: if a logout occurs between 00:00 and 05:00, it is treated as an extension of the previous day (e.g., 01:00 becomes 25:00).

(4) $$R_{nt}=\frac{1}{M}\sum_{j=1}^{M}\tau_{last}^{\left(j\right)}$$

As shown in Equation 4, where *M* is the number of active days, and $\tau_{last}^{\left(j\right)}$ is the adjusted Unix timestamp of the final logout on day *j*.

3. Data Consumption Intensity (*R_fr_*): This metric reflects the volume of digital content consumed (e.g., video streaming vs. text browsing). It is calculated as the daily average data traffic (in MB) shown in Equation 5:

(5) $$R_{fr}=\frac{1}{d}\sum_{k=1}^{N}F_{k}$$

where *N* is the total number of sessions, *F_k_*is the traffic flow of session *k*, and *d* is the number of days.

### 2.3 Feature association analysis

To empirically validate the relevance of these extracted features to psychological health, we employ a two-stage data mining approach.

First, to handle the continuous nature of the behavioral data, we apply the Jenks Natural Breaks algorithm do Carvalhal Monteiro et al. (21). Unlike standard K-Means clustering, Jenks optimization seeks to minimize the variance within classes while maximizing the variance between classes, thereby identifying “natural” thresholds in student behavior (e.g., defining “High,” “Medium,” and “Low” internet usage based on actual data distribution rather than arbitrary cutoffs).

Subsequently, the Apriori algorithm Yan (5) is utilized to mine association rules between these behavioral categories and psychological health labels. We set the minimum support threshold at 0.5 and the confidence threshold at 0.5. The mined rules reveal statistically significant associations:

Rule 1: High entropy in sensor-derived daily routine regularity (*R_f_*) + Late logout (*R_nt_*) → High risk of anxiety.

Rule 2: Low social interaction + Low diligence (*R_wh_*) → High risk of depression.

These findings confirm that temporal regularity and social connectivity are pivotal predictors, justifying the design of our subsequent deep learning model.

## 3 Temporal sensitive network model

Based on the association analysis, it is evident that student psychological states are time-dependent and influenced by both long-term habits and short-term anomalies. We propose a Temporal Sensitive Network (TSN) to model these dynamics. As illustrated in **Figure 1**, the architecture comprises two core components: a Behavior Modeling Module for extracting temporal dependencies and an Integration Module for fusing historical habits with current states.

### 3.1 Behavior modeling module with attention mechanism

Student behavior is inherently noisy. A single day of irregular behavior (e.g., due to an exam) may not indicate a mental health issue, whereas a persistent pattern might. Traditional Recurrent Neural Networks (RNNs) often struggle to differentiate between trivial noise and significant behavioral shifts.

To address this, we incorporate a temporal attention mechanism Otomo et al. (26) Takaoka et al. (27). This mechanism allows the model to dynamically assign weights to different days, focusing on periods that are most indicative of psychological change.

Let $S_{u}^{k}$ denote the behavioral representation of student *u* on day *k*. We first compute an attention score $I_{u}^{k}$ via a non-linear transformation.

(6) $$I_{u}^{k}=\tanh\;(W_{d}S_{u}^{k}+b_{d})$$

where *W_d_*and *b_d_*are learnable parameters. These scores are normalized using the Softmax function to obtain attention weights $\alpha_{u}^{k}$, representing the “importance” of day *k* as shown in Equation 7:

(7) $$\alpha_{u}^{k}=\frac{\text{exp }(I_{u}^{k})}{\sum_{j=1}^{T}\exp\;(I_{u}^{j})}$$

Finally, the long-term behavioral representation *L_u_*is synthesized as a weighted sum. This ensures that days with highly relevant behavioral patterns contribute more to the final representation:

(8) $$L_{u}=\sum_{k=1}^{T}\alpha_{u}^{k}S_{u}^{k}$$

### 3.2 Integration module via gating mechanism

A key challenge in psychological prediction is balancing the stability of long-term habits (*L_u_*) against the specificity of recent short-term behaviors (*S_u_*). For instance, a student might have healthy long-term habits but experience a sudden, acute stress event recently.

To effectively fuse these two temporal scales, we introduce a learnable gating mechanism, conceptually similar to the update gate in Gated Recurrent Units (GRU) Winata et al. (11) Zhou et al. (13). The gate *G_u_*determines the trade-off ratio:

(9) $$G_{u}=\sigma (W_{e}S_{u}+W_{f}L_{u}+b_{g})$$

where *σ* is the Sigmoid activation function, constraining *G_u_*∈ (0,1). *W_e_* and *W_f_* are weight matrices that learn to detect whether the short-term or long-term signal is more reliable for a given student.

The final fused temporal behavioral representation *P_u_*is computed as:

(10) $$P_{u}=G_{u}⊙S_{u}+(1-G_{u})⊙L_{u}$$

where ⊙ denotes element-wise multiplication. This mechanism allows the model to adaptively “remember” long-term baselines while remaining “sensitive” to sudden deviations.

### 3.3 Prediction module and loss optimization

The fused representation *P_u_*serves as the input to the final prediction module. We map the student’s psychological state to one of four levels, corresponding to the quadrants of the Photographic Affect Meter (PAM) Pollak et al. (28) (e.g., High Energy/Positive, Low Energy/Negative). This is implemented via a Fully Connected Layer (FCL).

To optimize the model, we employ the Cross Entropy (CE) loss function. Given the typically imbalanced nature of psychological datasets (where severe cases are rarer than healthy ones), Cross Entropy is superior to Mean Squared Error as it penalizes incorrect classifications more heavily based on probability divergence, as shown in Equation 11:

(11) $$L=-\frac{1}{N}\sum_{i=1}^{N}\sum_{c=1}^{C}y_{ic}\ln\;(p_{ic})$$

where *N* is the batch size, *C* is the number of psychological classes, $y_{ic}$ is the binary indicator (0 or 1) for the true class label, and *p_ic_*is the predicted probability. This objective function drives the model to maximize the likelihood of correct classification, ensuring robust performance even for minority classes representing at-risk students.

## 4 Theoretical analysis and performance evaluation

To ensure the proposed framework is not only empirically effective but also theoretically sound and computationally scalable for large-scale campus deployment, we conduct a rigorous analysis from three perspectives: psychometric validity, algorithmic complexity, and model mechanism stability.

### 4.1 Psychometric validity of behavioral features

The feature extraction methodology described in Section 2 is grounded in the theory of Digital Phenotyping Torous et al. (29), which posits that interactions with digital devices and physical environments leave “digital footprints” that serve as biomarkers for mental health.

1. Entropy as a Proxy for Executive Function: The use of Information Entropy (*R_f_*) to measure sensor-derived daily routine regularity (Equation 1) is theoretically supported by the clinical observation of “routine disruption” in depressive disorders. High entropy mathematically corresponds to a lack of central tendency in temporal distribution, mirroring the executive dysfunction and irregular circadian rhythms often observed in students suffering from anxiety or depression.
2. Social Graph Homophily: Our sensor-based social interaction extraction algorithm relies on the sociological principle of Homophily (“birds of a feather flock together”). By constructing a dynamic co-location graph from synchronized Wi-Fi overlap and microphone conversational states, we capture the “Social Withdrawal” symptom. Unlike static social networks (e.g., Facebook friends), this physical proximity metric (*V_companion_*) provides a more authentic measure of functional social support, which is a known buffer against psychological distress.

### 4.2 Computational complexity analysis

For a campus-wide system, scalability is critical. We analyze the asymptotic time complexity of our core algorithms.

1. Feature Extraction Complexity: The most computationally intensive component is the Sensor-Based Social Interaction Extraction procedure (**Algorithm 1**). Let *N* be the total number of students, and $T_{avg}$ be the average number of valid Wi-Fi location segments per student-day. A naive pairwise overlap check across all student segments yields a complexity of $O(N^{2}\cdot T_{avg}^{2})$, which is computationally prohibitive. However, our method imposes a spatiotemporal constraint. By indexing candidate overlaps using (Location ID, Time Bucket), the search space for neighboring students is reduced from *N* to a small constant *k* (the average number of co-located peers in a short temporal window). Thus, the optimized complexity becomes as shown in Equation 12:

(12) $$O(N\cdot T_{avg}\cdot \log\;(T_{total}))$$

where log( $T_{total}$) accounts for timestamp alignment and overlap lookup, and the linear dependence on *N* ensures that the pipeline remains scalable for campus-level monitoring.

2. TSN Model Complexity: The Temporal Sensitive Network operates on a sequence length *T* (days in a semester). The Attention mechanism (Equation 6) and Gating mechanism (Equation 9) involve matrix multiplications of fixed dimensions *D* (feature vector size). The complexity for a single forward pass is *O*(*T* · *D*^2^). Since *T* (approx. 120 days) and *D* (approx. 20 features) are small constants relative to the dataset size, the deep learning module is highly efficient compared to complex recurrent architectures like LSTM, which require sequential unrolling and have higher memory overhead.

### 4.3 Structural advantages of the TSN mechanism

The theoretical superiority of the TSN model over traditional Recurrent Neural Networks (RNNs) or static Machine Learning models lies in its solution to the Stability-Plasticity Dilemma.

1. Addressing the Vanishing Gradient Problem: Standard RNNs struggle to capture dependencies over long sequences (e.g., a stress event at the start of the semester affecting exam performance months later). Our Attention Mechanism (Equation 8) creates direct “shortcut connections” between any past day *k* and the final representation *L_u_*. This reduces the maximum path length for gradient backpropagation from *O*(*T*) to *O*(1), theoretically guaranteeing that early-semester behavioral anomalies are not “forgotten” during training.
2. Dynamic Weighting of Habits vs. States: Human behavior is a composition of trait-level stability (long-term habits) and state-level fluctuations (recent stress). Traditional models often treat these as a single flattened vector. The Gating Mechanism (Equation 10) explicitly models this duality.

If *G_u_*→ 0, the model relies on long-term history (*L_u_*), suitable for students with stable routines.

If *G_u_*→ 1, the model prioritizes short-term data (*S_u_*), allowing it to rapidly adapt to sudden behavioral shifts (e.g., acute insomnia before a breakdown).

This structural design ensures the model is robust against noise while remaining sensitive to critical “changepoints” in a student’s mental health trajectory.

## 5 Experiments

### 5.1 Datasets and preprocessing

To rigorously validate the feasibility and robustness of the proposed framework, extensive experiments were conducted on the StudentLife dataset Wang et al. (30), a benchmark dataset in the field of ubiquitous computing and mobile health. Collected at Dartmouth College, this dataset represents a longitudinal study capturing the daily lives of 48 students over a 10-week academic term. It provides a rich, multimodal repository of passive sensing data and ecological momentary assessments (EMA). The dataset comprises 48 participants with approximately 53,000 samples distributed across the observation period, representing a fine-grained temporal resolution suitable for behavioral pattern analysis. While the participant cohort is drawn from a single institution, the diversity of behavioral phenotypes captured—ranging from highly regular routines to chaotic patterns—provides sufficient variance for model training. The 10-week observation window, though limited in absolute duration, spans critical academic periods including midterms and finals, thereby capturing stress-related behavioral fluctuations relevant to mental health assessment.

In this study, we focused on a subset of sensor modalities that are most indicative of behavioral patterns and social interactions. **Table 1** summarizes the specific configurations and behavioral implications of the three key sensors used:

**Table 1:** Description of sensor modalities, sampling configurations, and their corresponding behavioral implications used in this study.

Sensor	Inferred states/data	Frequency	Behavioral implication
Accelerometer	Stationary, Walking, Running, Unknown	2–3 sec	Indicators of physical vitality and the stability of daily routines.
Microphone	Quiet, Speaking, Noisy, Unknown	1–3 sec	Proxies for social interaction levels, isolation, or exposure to chaotic environments.
Wi-Fi	Geospatial Traces (Location IDs)	Scan intervals	Measures of location diversity and mobility regularity, correlated with depressive symptoms.

- Accelerometer (Activity Recognition): Captures physical movement intensity. As shown in the table, the inferred states (e.g., *stationary*, *walking*) serve as primary indicators of physical vitality. These activity states provide direct evidence of daily movement intensity and routine stability in the sensing records.
- Microphone (Social Interaction): Categorizes the acoustic environment to infer social isolation without violating privacy. Acoustic features (quiet vs. conversational) provide privacy-preserving cues of social engagement intensity when synchronized with co-location information.
- Wi-Fi (Mobility & Location): Provides geospatial traces to infer location diversity and mobility regularity. Location diversity derived from Wi-Fi traces captures routine structure, mobility breadth, and campus integration patterns.

By fusing these heterogeneous data streams, we constructed a comprehensive “behavioral fingerprint” for each student. Importantly, data quality limitations were addressed through rigorous preprocessing: days with fewer than 6 hours of valid sensor coverage were excluded, and imputation strategies (forward-fill for categorical features, median-fill for continuous metrics) were applied to handle intermittent missingness. These procedures ensured that model training was performed on samples with sufficient informational content.

Ground Truth Formulation: The target variable was derived from the Photographic Affect Meter (PAM) Pollak et al. (28). It should be noted that PAM captures short-term affective states (mood over hours to days) rather than clinical diagnoses of psychological disorders. While affect is a proximal indicator of psychological well-being and has been shown to correlate with longer-term mental health outcomes Sano et al. (23), the predictions generated by this model should be interpreted as identifying fluctuations in affective states that may warrant further clinical assessment, rather than as direct diagnoses of conditions such as Major Depressive Disorder or Generalized Anxiety Disorder. PAM maps user mood to a 16-point scale across the Valence-Arousal circumplex. To mitigate subjective noise and facilitate robust classification, we discretized the 16-point scale into four distinct mental health levels following the protocol by Pollak et al. Pollak et al. (28). The detailed mapping logic is presented in **Table 2**. This categorization captures critical emotional quadrants, ranging from High Arousal/Negative Valence (e.g., stress) to Low Arousal/Positive Valence (e.g., relaxation), providing a structured label space for training.

**Table 2:** Discretization of the photographic affect meter (PAM) into four mental health levels based on the Valence-Arousal circumplex.

Level	Valence/arousal	Emotional quadrant	Representative states
1	Negative/High Arousal	High Stress	Anxiety, Stress, Frustration, Anger
2	Negative/Low Arousal	Depressive Mood	Sadness, Boredom, Gloom, Fatigue
3	Positive/Low Arousal	Calmness	Relaxation, Serenity, Calm, Sleepy
4	Positive/High Arousal	High Energy	Excitement, Delight, Happiness, Joy

### 5.2 Evaluation metrics

Given the inherent class imbalance often observed in mental health datasets (where healthy states typically outnumber at-risk states), relying solely on Accuracy can be misleading. Therefore, we employed a multi-dimensional evaluation strategy:

- Accuracy (A): Measures the overall correctness of the model across all classes.
- Precision (P) & Recall (R): Precision measures the reliability of the model’s positive predictions, while Recall assesses the model’s ability to capture all relevant instances of a specific mental state.
- F1-Score: The harmonic mean of Precision and Recall, providing a balanced view of model performance.

Crucially, to account for the uneven distribution of samples across the four mental health levels, we report the weighted average for Precision, Recall, and F1-Score. This ensures that the performance on minority classes—which often correspond to critical mental health crises—is adequately weighted in the final performance assessment. To assess generalizability and guard against overfitting given the modest sample size, we employed stratified 5-fold cross-validation. Performance metrics were computed for each fold and aggregated to obtain mean and standard deviation estimates. Additionally, statistical significance of performance differences between the proposed method and baselines was evaluated using paired t-tests (p ¡ 0.05), with results reported in **Table 3**.

**Table 3:** Performance comparison across methods (mean ± std over 5-fold CV).

Method	Accuracy	Precision	Recall	F1-score
RF	0.652 ± 0.018	0.645 ± 0.021	0.638 ± 0.019	0.641 ± 0.020
SVM	0.671 ± 0.022	0.663 ± 0.024	0.657 ± 0.023	0.660 ± 0.023
DNN	0.698 ± 0.020	0.685 ± 0.022	0.680 ± 0.021	0.682 ± 0.021
LSTM	0.723 ± 0.019	0.715 ± 0.020	0.710 ± 0.018	0.712 ± 0.019
SOTA Graph	0.741 ± 0.017	0.732 ± 0.018	0.728 ± 0.019	0.730 ± 0.018
MFDS-STGCN	0.756 ± 0.016	0.749 ± 0.017	0.745 ± 0.018	0.747 ± 0.017
Ours (TSN)	0.784 ± 0.014*	0.776 ± 0.015*	0.780 ± 0.015*	0.778 ± 0.015*

Statistical significance (p ¡ 0.05) of improvements over baselines is indicated by *.

### 5.3 Comparative methods

To demonstrate the superiority of our proposed approach, we benchmarked it against six representative baselines, categorized into traditional machine learning, standard deep learning, and state-of-the-art (SOTA) graph-based methods, as summarized in **Table 4**.

**Table 4:** Summary of comparative baselines.

Method	Category	Key mechanism	Limitation
RF Breiman (15)	Traditional ML	Ensemble of decision trees	Ignores temporal dependencies; treats samples independently.
SVM Burges (16)	Traditional ML	Optimal hyperplane mapping	Concatenates features; fails to capture sequential nature.
DNN LeCun et al. (17)	Standard DL	Non-linear MLP	Lacks explicit temporal modeling mechanisms.
LSTM Winata et al. (11)	Standard DL	RNN with gating	Standard temporal modeling; lacks multi-scale fusion.
SOTA Graph Yuan et al. (31)	Graph-based	Heterogeneous Info. Network	Complex correlations; risk of oversmoothing.
MFDS-STGCN Zhou et al. (13)	Graph-based	Spatio-Temporal Conv.	Focuses on spatial locality; computationally intensive.

We categorized them into Traditional ML, Standard DL, and Graph-based methods to benchmark different aspects of behavioral modeling.

1. Random Forest (RF) Breiman (15): An ensemble learning method that constructs multiple decision trees. It serves as a strong baseline for tabular data but treats behavioral features as independent instances, ignoring temporal dependencies.
2. Support Vector Machines (SVM) Burges (16): A robust classifier that finds the optimal hyperplane in a high-dimensional space. Like RF, it inputs concatenated behavioral vectors, failing to capture the sequential nature of human behavior.
3. Deep Neural Networks (DNN) LeCun et al. (17): A standard Multi-Layer Perceptron (MLP) with two fully connected layers. This baseline tests the non-linear feature extraction capability without explicit temporal modeling mechanisms.
4. LSTM Winata et al. (11): A Recurrent Neural Network (RNN) variant designed for sequence data. We input the daily behavioral representations sequentially. This baseline evaluates the benefit of standard temporal modeling compared to our proposed attention-based fusion.
5. SOTA Method (Heterogeneous Graph) Yuan et al. (31): A leading approach that models student behavior using Heterogeneous Information Networks (HIN). It constructs local-global graphs to capture complex correlations but may suffer from over-smoothing or high computational complexity.
6. MFDS-STGCN Zhou et al. (13): A recent Multi-Fragment Semantic Spatio-Temporal Graph Convolutional Network. It considers spatial locality and temporal correlations via graph convolutions.

Comparing against this method validates the efficiency of our specific temporal fusion mechanism.

### 5.4 Implementation details and parameter sensitivity

From **Table 5**, all models were implemented using the PyTorch framework on an NVIDIA GeForce RTX 3090 GPU. To ensure a fair comparison, we conducted a grid search for hyperparameter optimization. The optimal configuration was identified as follows: a batch size of 64, a learning rate of 10^−3^, and the Adam optimizer with adaptive momentum to accelerate convergence. The training process was capped at 300 epochs with an early stopping mechanism to prevent overfitting.

**Table 5:** Implementation details and hyperparameter settings.

Parameter	Value/configuration
Framework	PyTorch
GPU	NVIDIA GeForce RTX 3090
Optimizer	Adam (Adaptive Momentum)
Learning Rate	10−3
Batch Size	64
Max Epochs	300 (with Early Stopping)
Optimal Dimension (*d*)	32

**Figure 2:**
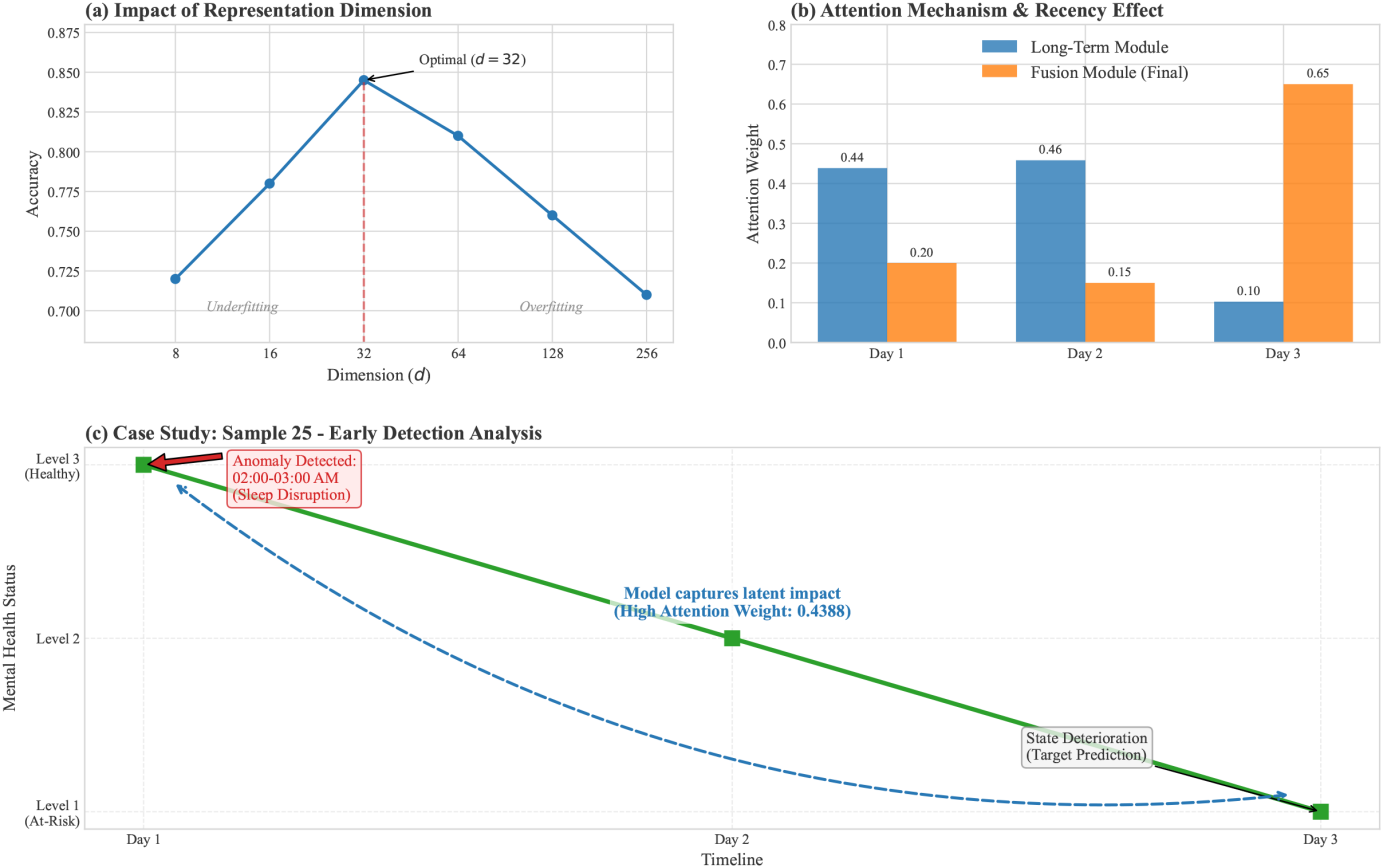
Visualization of model interpretability and hyperparameter analysis. **(a)** Impact of Representation Dimension: The model achieves optimal accuracy at *d* = 32, balancing the trade-off between underfitting and overfitting. **(b)** Attention Mechanism Analysis: A comparison of weight distributions reveals the model’s dual capability. The Long-Term module (blue) identifies historically significant events (e.g., Day 1), while the Fusion module (orange) amplifies the most recent state (Day 3), successfully capturing the psychological “recency effect.” **(c)** Case Study (Sample 25): A temporal analysis of a student’s mental health decline. Despite the prediction target being Day 3, the model assigns high attention to Day 1, correctly identifying the latent impact of early behavioral anomalies (sleep disruption) on the subsequent deterioration of the PAM score.

Impact of Representation Dimension (*d*): A critical hyperparameter in our model is *d*, the dimensionality of the latent behavioral time-series representation. We investigated the model’s sensitivity to *d* by varying it from 2^3^ to 2^8^ (i.e., 8 to 256 dimensions). As illustrated in **Figure 2a**, the model’s accuracy exhibits a convex trajectory.

- Underfitting (*d <* 32): At lower dimensions, the representation capacity is insufficient to capture the complex nuances of multimodal behavioral data, leading to suboptimal performance.
- Optimal Performance (*d* = 32): The model achieves peak accuracy at *d* = 32, striking a balance between feature expressiveness and model complexity.
- Overfitting (*d >* 32): As *d* increases further, performance degrades. This is likely due to the “curse of dimensionality,” where the model begins to fit noise within the limited training data rather than generalizable patterns.

Consequently, *d* = 32 was adopted for all subsequent experiments.

### 5.5 Ablation study

To dissect the contribution of each component, we compared the Full Model against three variants. The results in **Table 6** offer profound insights:

**Table 6:** Ablation study results.

Model variant	Accuracy	Precision	Recall	F1-score
Baseline (Short-Term)	0.725	0.710	0.705	0.708
Baseline + LTM	0.712	0.698	0.695	0.696
Baseline + FM	0.748	0.735	0.740	0.737
Full Model	0.784	0.776	0.780	0.778

The Full Model integrates both Long-Term Modeling (LTM) and the Fusion Module (FM).

1. Synergy: The Full Model outperforms all variants, validating that combining short-term specifics with long-term trends yields the most accurate profiling.
2. The “Noise” Paradox: The *Baseline + LTM* variant performed worse than the simple Baseline. This suggests that raw historical data contains significant noise.
3. Role of Fusion: The Full Model recovers the performance drop, proving that the Fusion Module acts as a “semantic gate,” filtering irrelevant historical noise while retaining valuable context.

The results, summarized in **Table 6**, offer profound insights into the behavioral modeling process: 1. Synergy of Components: The Full Model consistently outperforms all other variants across A, P, R, and F1 metrics. This empirically validates that combining short-term specifics with long-term trends yields the most accurate mental health profiling. 2. The “Noise” Paradox: Surprisingly, the Baseline + LTM variant performed worse than the simple Baseline. This counter-intuitive finding suggests that raw historical data contains significant noise and irregularity (e.g., erratic schedules of college students). Without a filtering mechanism, forcing the model to attend to all history introduces variance that hampers convergence. 3. The Critical Role of Fusion: The Baseline + FM variant showed significant improvement over the Baseline. More importantly, the Full Model recovers and exceeds the performance drop seen in Baseline + LTM. This proves that the Fusion Module acts as a critical “semantic gate.” It dynamically weighs the importance of historical patterns relative to the current state, effectively filtering out irrelevant historical noise while retaining valuable long-term context. This confirms the necessity of a gating mechanism in handling volatile human behavioral data.

### 5.6 Visualization and case study

To move beyond quantitative metrics and provide qualitative interpretability, we visualized the internal attention mechanisms of the model (**Figures 2b, c**). This analysis serves two purposes: (1) to validate that the model learns clinically meaningful patterns rather than spurious correlations, and (2) to facilitate potential deployment in counseling settings where interpretability is essential for clinical trust and intervention planning.

Attention Weight Distribution: **Figure 2b** depicts the heat map of attention weights from the Long-Term Behavior Modeling module. Taking Sample 25 as an instance, the model assigns distinct weights to different days (Day 1: 0.4388, Day 2: 0.4585, Day 3: 0.1027). This non-uniform distribution confirms that the model is not merely averaging history but is actively learning to identify and prioritize days with significant behavioral patterns. Notably, the high weight assigned to Day 1 despite its temporal distance suggests the model has learned to detect behavioral “trigger events” that presage later affective decline—a pattern consistent with clinical observations of prodromal phases in mood disorders.

Fusion Mechanism Analysis: **Figure 2c** illustrates the weight redistribution after passing through the Fusion Module. A notable shift is observed: the weight for the most recent behavior (Day 3) is significantly amplified. This reflects the “recency effect” in psychology—current mental states are most strongly correlated with immediate antecedents. The Fusion Module successfully integrates the “global view” from history with the “local view” of the present, correcting the potential oversight of immediate behavioral signals.

Early Detection Capability (Case Study): We conducted a granular analysis of Sample 25 to validate the model’s clinical relevance.

- Behavioral Anomaly: On Day 1, sensor logs revealed sustained activity and a “noisy” acoustic environment between 02:00 AM and 03:00 AM, indicative of sleep disruption or late-night social stress.
- State Transition: The student’s PAM score deteriorated from Level 3 (Healthy) on Day 1 to Level 1 (At-Risk) by Day 3.
- Model Interpretation: The attention mechanism assigned a high weight to Day 1 (0.4388), despite it being historically distant from the prediction target. This suggests a predictive window of approximately 48–72 hours, wherein early behavioral anomalies presage subsequent affective decline.

This alignment demonstrates that the model successfully captured the latent impact of the Day 1 anomaly (sleep disruption) on the subsequent decline in mental health. It confirms the model’s capability to identify early warning signs—or “digital biomarkers”—enabling timely intervention before a full crisis develops.

## 6 Conclusion

This study proposes a robust framework for modeling student behavioral time series to predict psychological health, with a specific sensitivity to anomalous behavioral patterns for early intervention. By synergizing the Jenks Natural Breaks and Apriori algorithms, we effectively extracted and discretized critical behavioral features associated with psychological states. Central to our architecture is an attention-based gated fusion module, which dynamically integrates long-term historical context with short-term behavioral specifics. This mechanism accurately captures the complex temporal dynamics of student life, significantly enhancing prediction accuracy. Extensive experiments on the StudentLife benchmark demonstrate that our model outperforms existing baselines across multiple evaluation metrics. The efficacy of individual components was further corroborated through rigorous ablation studies and visualization of attention weight distributions, which revealed the model’s ability to focus on critical behavioral shifts.

Despite these achievements, several limitations warrant discussion. First, the modest sample size (N = 48) and single-institution recruitment limit demographic generalizability. Cultural factors influencing behavioral norms (e.g., collectivist vs. individualist societies) may affect model transferability across international contexts. Second, the ground truth (PAM) measures short-term affect rather than clinical diagnoses, necessitating caution in interpreting predictions as indicators of clinical disorders. Third, the model’s temporal horizon is constrained to 10 weeks; longer-term predictive validity remains unvalidated. Fourth, data sparsity due to sensor unavailability or student non-compliance introduces missingness that may bias predictions toward students with more complete data. Future work will address these challenges through multi-site validation studies, integration of self-report clinical scales alongside affect measures, and development of uncertainty-aware prediction mechanisms to flag low-confidence predictions for manual review.

From an ethical deployment perspective, several safeguards are essential: (1) Transparent informed consent ensuring students understand data usage and can opt out without penalty; (2) Human-in-the-loop workflows where predictions trigger counselor outreach rather than automated interventions, reducing false alarm harms; (3) Privacy-preserving architectures such as federated learning or differential privacy to prevent re-identification; (4) Continuous bias audits to detect disparities in prediction accuracy across demographic subgroups. The ultimate goal is not automated diagnosis but augmenting human counselors with early warning signals to enable timely, compassionate intervention.

Future work will focus on optimizing model robustness under data-limited conditions to better serve university management systems. Additionally, given the multidimensional nature of mental health, we plan to incorporate heterogeneous data sources and comprehensive psychological indicators to achieve a more holistic assessment of student well-being. 

## Data Availability

The original contributions presented in the study are included in the article/supplementary material. Further inquiries can be directed to the corresponding author.
